# Genetic landscape of breast cancer and mutation tracking with circulating tumor DNA in Chinese women

**DOI:** 10.18632/aging.202888

**Published:** 2021-04-23

**Authors:** Yinghao Wang, Lizhi Lin, Linhong Li, Jialiang Wen, Yili Chi, Rutian Hao, Xuanxuan Dai, Yizuo Chen, Duping Huang, Yili Zhou, Jie You, Zhiqiang Ye, Hao Chen, Lingli Jin, Danxiang Chen, Fan Yang, Erjie Xia, Xueyan Ma, Fengyu Guo, Yunguang Tong, Min Zheng, Ouchen Wang

**Affiliations:** 1 Department of Breast Surgery, The First Affiliated Hospital of Wenzhou Medical University, Wenzhou 325000, P.R. China; 2 Omigen, Inc., Hangzhou 310000, P.R. China; 3 Department of Breast Surgery, The Second Affiliated Hospital and Yuying Children’s Hospital of Wenzhou Medical University, Wenzhou 325000, P.R. China

**Keywords:** breast cancer, digital molecular identifier, circulating tumor DNA, mutation landscape, Chinese women

## Abstract

Considerable efforts have been devoted to exploring the breast cancer mutational landscape to understand its genetic complexity. However, no studies have yet comprehensively elucidated the molecular characterization of breast tumors in Chinese women. This study aimed to determine the potential clinical utility of peripheral blood assessment for circulating tumor-derived DNA (ctDNA) and comprehensively characterize the female Chinese population’s genetic mutational spectrum. We used Omi-Seq to create cancer profiles of 273 patients enrolled at The First Affiliated Hospital of Wenzhou Medical University. The gene landscape results indicate *PIK3CA* and *TP53* as the most frequently detected genes, followed by *ERBB2,* in Chinese breast cancer patients. The accuracy of *ERBB2* copy number variations in tissue/formalin-fixed and paraffin-embedded samples was 95% with 86% sensitivity and 99% specificity. Moreover, mutation numbers varied between different molecular cell-free DNA subtypes, with the basal-like patients harboring a higher number of variants than the luminal patients. Furthermore, ratio changes in the max ctDNA allele fraction highly correlated with clinical response measurements, including cancer relapse and metastasis. Our data demonstrate that ctDNA characterization using the Omi-Seq platform can extend the capacity of personalized clinical cancer management.

## INTRODUCTION

Breast cancer is one of the most common cancers and the second leading cause of cancer-related deaths in women worldwide [1, 2]. The incidence of breast cancer in China is approximately 40/100,000, and 70,000 people have died because of breast cancer in 2015 [3]. Breast cancer is a heterogeneous disease, resulting in complex clinical characteristics and molecular subtypes. Thanks to the development of high-throughput sequencing technology, breast cancer is composed of at least four molecular-specific diseases with different characteristics, clinical behavior, and treatment responses. These intrinsic molecular subtypes are defined as: basal-like, human epidermal growth factor receptor 2 (HER2) enrichment, and luminal A and B subtypes [4, 5]. Because of intrinsic molecular heterogeneity, response to treatment differs between different molecular subtypes. Hence, accurately characterizing the tumor molecular subtype is necessary to guide optimal treatment decisions and determine patient prognosis [6]. Despite significant advances in treatment, metastasis remains the leading cause of breast cancer-related death; this is because of the associated tumor heterogeneity, owing to clonal evolution, limiting therapeutic efficacy and response time [7–9]. Therefore, evaluating tumor clonal heterogeneity is of significant value for breast cancer patients [10]. However, imaging examination, the main traditional diagnostic method for breast cancer, cannot determine the patient’s gene mutations and tumor clonal heterogeneity and cannot facilitate the development of individualized treatment regimens. Of note, next-generation sequencing (NGS) technologies and bioinformatics tools have increased the understanding of breast cancer molecular heterogeneity, thereby providing an avenue for the identification of targeted mutations and subsequent personalized patient treatment strategies [11].

From the first detection of extracellular nucleic acids in peripheral blood in 1948 to the detection and quantification of cancer-related DNA mutations in 1994, liquid biopsies are continually evolving in the field of patient care [12]. Nucleic acids circulating in peripheral blood and originating from tumor masses are designated circulating tumor DNA (ctDNA), and their detection serves as a framework for the non-invasive detection of genomic alterations. Moreover, plasma-derived ctDNA is suitable for real-time monitoring of therapy responses because of its short lifetime (approximately 2 hours) [13]. Several studies have explored and evaluated the potential clinical value of ctDNA in cancer management, including for early detection, auxiliary evaluation of minimal residual disease, treatment monitoring, and drug resistance assessment [14–17].

Although deep sequencing platforms have been widely adopted for investigating subpopulations in complex biological samples, certain inherent limitations related to the relatively low yield of ctDNA and the errors introduced during sample preparation and sequencing interfere are their limitations [18]. Moreover, although molecular identifiers can theoretically limit the prevalence of these associated errors, certain challenges remain. First, synthesizing double-stranded adapters with randomized molecular identifiers is difficult. Second, owing to the difficulty of generating high-quality single molecular identifier (SMI) adaptors, the ligation efficiency may be compromised and require large amounts of input DNA [19]. The Omi-Seq platform (developed by Omigen, Inc.) may address some of these challenges as it calculates digital molecular identifiers (DMIs) using a standard adapter pool with defined barcodes that can readily synthesize high-quality sequence reads.

In the present study, we used Omi-Seq to create cancer profiles of female Chinese breast cancer patients. The resulting ctDNA genomic mutational profiles were then compared between patients with early and metastatic cancer and different pathological subtypes. This study’s primary aim was to define the potential clinical utility of peripheral blood ctDNA detection and analysis and characterize the genetic mutation spectrum within the female Chinese breast cancer population.

## RESULTS

### Patients’ characteristics

A total of 273 breast cancer patients were enrolled in the study (Supplementary Table 1). All patients were women, and the median age was 52 years. The patients were categorized based on tumor subtype, with 77 luminal A (28.2%), 74 luminal B (27.1%), 67 HER2+ (24.5%), and 45 basal-like (16.5%) patients included. Some of the patients had received monotherapy with an aromatase inhibitor (AI), while others had been administered combination AI or fulvestrant and CDK4/6 inhibitor therapy. However, no patients had received prior poly ADP ribose polymerase (PARP) inhibitor therapy.

### The ctDNA mutational landscape in Chinese women with breast cancer

**Figure 1:**
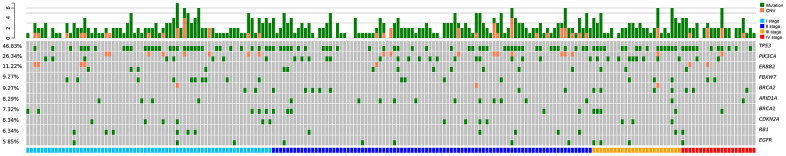
**Low-frequency somatic mutations detected in DMI-tagged ctDNA from Chinese breast cancer patients.** Mutational profiles derived from DMI-tagged ctDNA from stage I (blue), II (deep blue), III (yellow), and IV (red) breast cancers. Each column represents one patient. Different colors represent different types of mutations. Green and orange colors represent mutations and CNV, respectively. Each row represents one gene. The top bar graph denotes the number of mutations detected in each patient. The sidebar represents the proportion of patients with a mutation in a certain gene. CNV, copy number variation; ctDNA, circulating tumor-derived DNA; DMI, digital molecular identifier.

High-frequency molecular variants were analyzed in pairs of tumor tissue and plasma. The consistency, defined as harboring at least one common gene mutation site, was evaluated between the tumor tissue and paired plasma samples. The consistency values between tissue and plasma samples in stage I, II, III, and IV breast cancers were 80% (32/40), 80% (36/45), 100% (13/13), and 100% (3/3), respectively. The top three most prevalent mutated genes were as follows: *PIK3CA* (tissue: 42.31%, plasma: 24.04%), *TP53* (tissue: 32.69%, plasma: 43.27%), and *ERBB2* (tissue: 19.23%, plasma: 14.42%). The top three variants were *PIK3CA* p.H1047R (c.3140A>G), *PIK3CA* p.E545K (c.1633G>A), and *PIK3CA* p.E542K (c.1624G>A) (Figure 1). Our findings were consistent with those reported by Andre et al [20].

**Figure 2:**
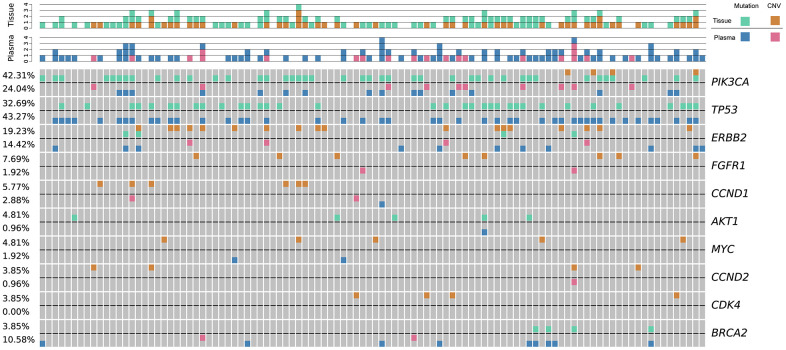
**The genetic landscape of tissue DNA and plasma ctDNA alterations in Chinese breast cancer patients.** Green and orange colors represent mutations, and CNV detected in tissue samples, respectively; blue and pink colors represent mutations, and CNV detected in cfDNA samples, respectively. Each column represents one patient. Each row represents one gene. The top bar denotes the number of mutations detected in each patient. The sidebar represents the proportion of patients with a mutation in a certain gene. CNV, copy number variation; ctDNA, circulating tumor-derived DNA; cfDNA, cell-free DNA.

Next, matched DMI-tagged cell-free DNA (cfDNA) was sequenced using the Omigen 101 genes panel kit at a targeted sequencing depth of 40,000×–50,000×. The mutation profile was derived from 205 detected female Chinese patients’ cfDNA with an overall mutation detection rate of 94.15% (193/205; Figure 2). The allele fraction (AF) detected in our cohort ranged from 0.05%–32.48%. Some of these mutations were actionable, including SNVs, such as *PIK3CA* p.H1047L, p.E545K, and p.E542K, *BRCA1/2* loss-of-function oncogenic mutation, and copy number mutations, such as *ERBB2* amplification. No mutation was detected from the panel of the remaining 12 patients. Moreover, the patients with negative cfDNA tests were primarily in stage I or II of disease, including six patients in stage I and four in stage II (Figure 2).

These data suggest that somatic mutations with frequencies as low as 0.05% could be detected in DMI-tagged cfDNA of early-stage breast cancer patients with a targeted sequencing depth of 40,000×–50,000×.

### Mutation landscape across different molecular subtypes

**Figure 3:**
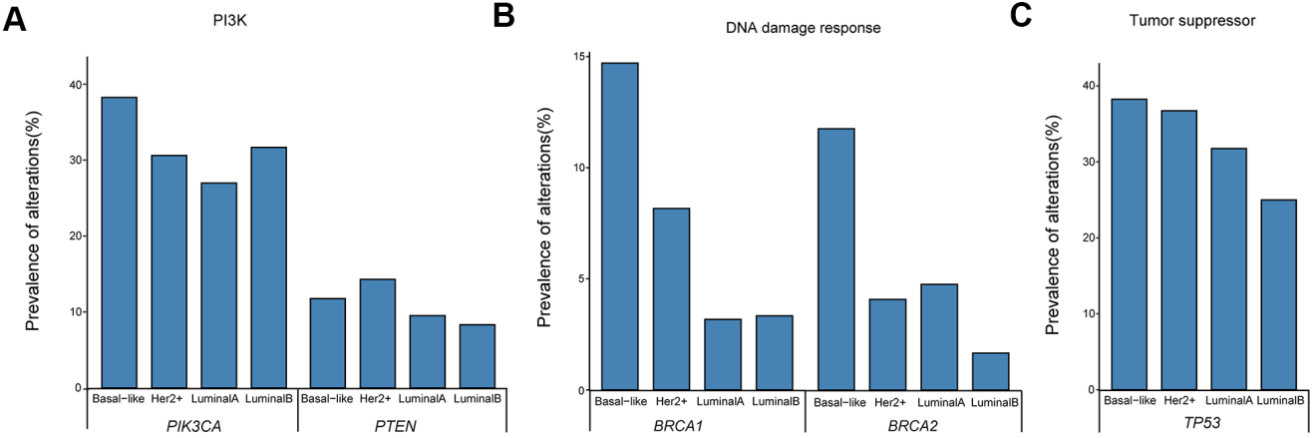
Prevalence of ctDNA oncogenic mutations in the (**A**) PI3K pathway and loss-of-function mutations in (**B**) DNA damage response and (**C**) tumor suppressor pathways. ctDNA, circulating tumor-derived DNA.

The distribution of mutations in patients was further analyzed based on four molecular subtypes. The prevalence of changes in cancer-related genes/pathways was compatible with that reported in previous studies of treated advanced breast malignancies, including frequent oncogenic mutations in the PI3K pathway and loss-of-function mutations in the DNA damage response and tumor suppressor pathways [21, 22]. Frequently varied genes in all four molecular subtypes are presented in Figure 3. The *PIK3CA* detection rates in basal-like (38.24%, 13/34) were higher than in luminal B (31.67, 19/60), HER2+ (30.61%, 15/49), and luminal A (26.98 %, 17/63) breast cancers (Figure 3A). Meanwhile, *PTEN* mutations were more frequently detected in HER2+ and basal-like subtypes and were less common in the luminal type, with detection rates of 14.29% (7/49), 11.76% (4/34), 9.52% (6/63), and 8.33% (5/60), respectively (Figure 3A). *BRCA1/2* mutations were predominantly detected in basal-like patients (14.71%, 11.76%; Figure 3B), while *TP53* mutations were frequently detected in all four molecular subtypes (38.24%, 36.73%, 31.75%, and 25% for basal-like, HER2+, luminal A, and luminal B, respectively; Figure 3C).

### cfDNA yield and ctF (highest cfDNA allele frequency) correlate with cancer stage

**Figure 4:**
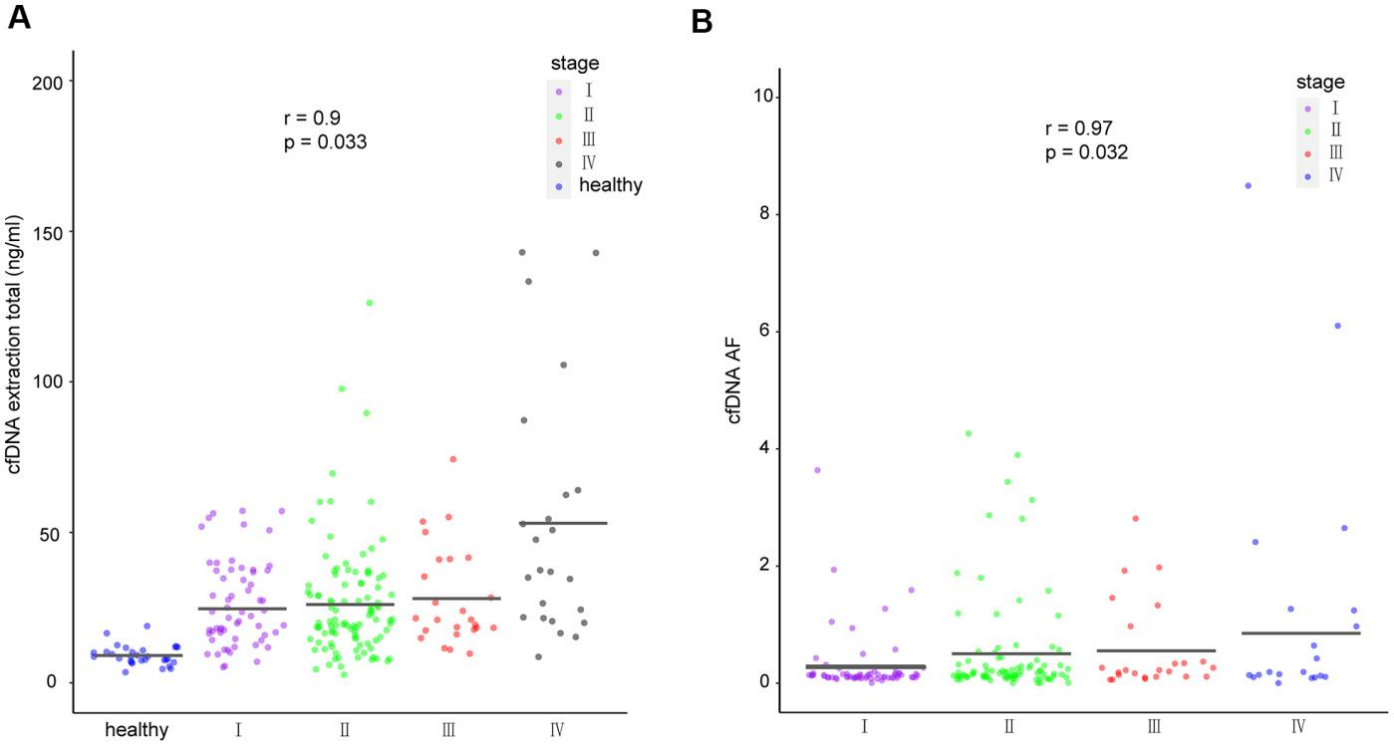
**cfDNA in healthy individuals and breast cancer patients.** (**A**) Amount of cfDNA extracted from all healthy individuals and cancer patients of different stages. (**B**) Mutant allele frequency of cfDNA detected in patients with different cancer stages. The means for each group are represented by the black lines in each column. cfDNA, cell-free DNA.

To investigate the correlations between cfDNA and corresponding clinical characteristics, we analyzed cfDNA yield in healthy participants and breast cancer patients with different disease stages. Considering that tumors generate a certain amount of free nucleotides into the peripheral blood, the cfDNA yield could represent the amount of released tumor DNA in the patient’s body [23, 24]. We found that cfDNA was significantly associated with disease stage (p = 0.033, r = 0.9; Figure 4A). Meanwhile, ctF, defined as the highest cfDNA allele frequency in patients with multiple alterations, was found to be significantly associated with disease stage (p = 0.032, r = 0.97; Figure 4B).

### Number of ctDNA mutations in different molecular subtypes and disease stages

**Figure 5:**
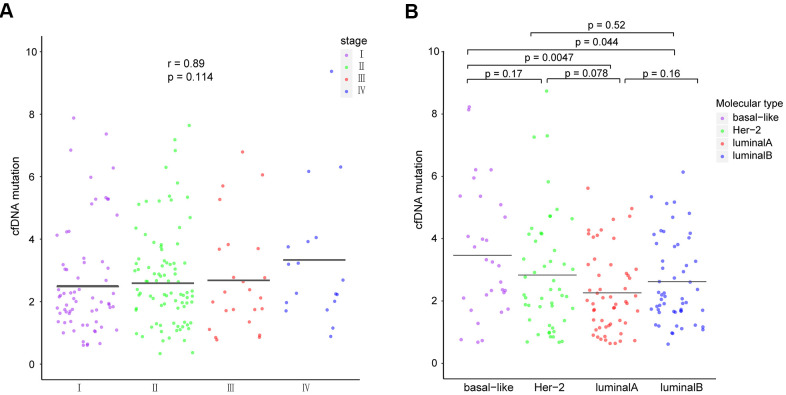
Comparison of ctDNA among patients with different (**A**) breast cancer stages and (**B**) molecular subtypes. The means of each group are represented by black lines in each column. ctDNA, circulating tumor-derived DNA.

Cancers with higher tumor mutational burdens (TMBs) are at a higher risk of being recognized by immune cells [25]. Hence, we used the total number of ctDNA non-synonymous coding mutations detected per megabase to calculate TMB and detected the highest TMB in the basal-like subtype, achieving statistical significance in comparison with the luminal subtypes (luminal A: p = 0.0047, luminal B: p = 0.044; Figure 5B). Our data also shows that the total number of ctDNA mutations positively correlates with the breast cancer stage (p = 0.114, r = 0.89; Figure 5A), although this result was not statistically significant.

### NGS detection using tissue samples better reflects ERBB2 copy number amplification than using blood samples

We next sought to explore the application of NGS detection for predicting *ERBB2* copy number amplification in breast cancer patients. To this end, we applied a 101 gene panel for detecting *ERBB2* copy number amplification in 123 tissues and 202 blood samples. Immunohistochemistry (IHC) and Fluorescence *in situ* hybridization (FISH) were performed simultaneously. We then analyzed and compared the *ERBB2* copy number amplification results obtained by all the methods. The NGS results for tissue samples were highly consistent with IHC and FISH, with a sensitivity of 86%, a specificity of 99%, and accuracy of 95% (ratio of *ERBB2* duplication region ≥75%, The duplication of *ERBB2* is identified when the proportion of amplified *ERBB2*'s exon region greater than or equal to 0.75). However, the results of NGS for blood samples were relatively inconsistent with those of IHC and FISH results, presenting a sensitivity of only 5.4%, a specificity of 97%, and accuracy of 71% (Supplementary Table 2).

For samples with an IHC test result of 2+, an additional FISH test was required to determine the final *ERBB2* expression. We analyzed the FISH and NGS results for tissue samples simultaneously to evaluate the consistency between the two detection methods in IHC 2+ samples. We obtained 100% consistency in 12 tissue samples, implying the perfect accuracy of NGS for the detection of *ERBB2* copy number amplification in IHC 2+ samples (Supplementary Table 3). Our findings, therefore, support the application of NGS for the detection of *ERBB2* copy number amplification in tissue samples, particularly when combined with NGS and IHC, which would serve to improve the accuracy of detecting *ERBB2* copy number amplification.

### Dynamic monitoring and prognosis assessment

The application of ctDNA for dynamic monitoring and prognostic assessment of tumor patients was also explored. We focused on serial samples of 15 patients who underwent two or more ctDNA tests.

**Figure 6:**
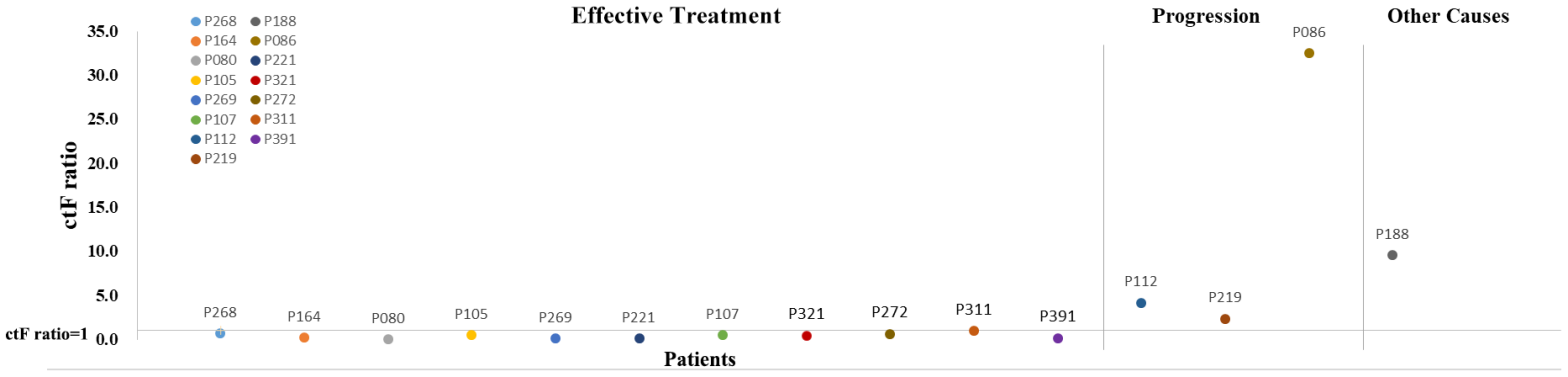
**Scatter diagram for ctF ratio of 15 patients with two or more cfDNA tests.** The horizontal line on the abscissa indicates that the ctF ratio is 1. Each dot represents one patient. cfDNA, cell-free DNA; ctF, cfDNA allele frequency.

Eleven patients presented with decreased ctF ratio (ctF ratio: ctF2/ctF1, ctF1 refers to the highest AF in the first test, and ctF2 refers to the highest AF in the second test) (Figure 6, Supplementary Table 4), which is consistent with their imaging findings: 10 patients had significantly shrunken tumor size, and 1 patient had no significant increase in tumor size. Meanwhile, four patients had increased ctF ratios during or after treatment, 3 of whom P112, P219, and P86, had metastasis or relapse. The remaining patient required continued follow-up and observation to determine the clinical outcome.

**Figure 7:**
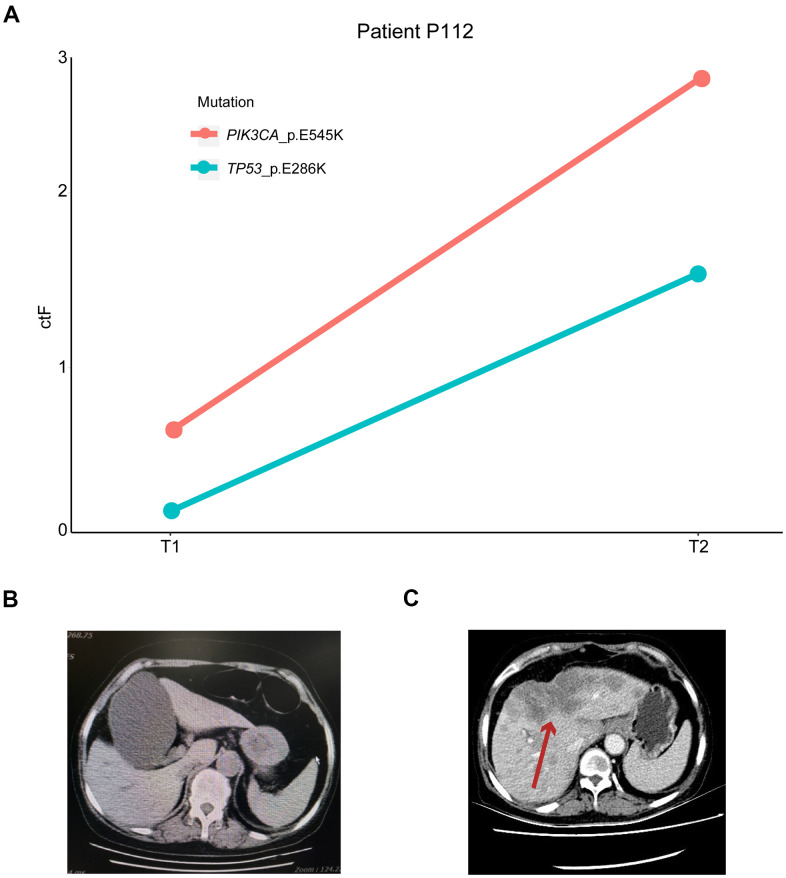
**Mutation tracking in metastasis and recurrence patients.** (**A**) ctDNA mutation tracking in patient P112. Computed tomography scan of P112 patient on (**B**) T1 and (**C**) T2 follow-ups. The red arrow indicates the patient’s metastatic foci. ctDNA, circulating tumor-derived DNA.

Patient P112 had locally advanced breast cancer, which was first diagnosed in December 2015, and received chemotherapy, endocrine therapy, and radiotherapy. The disease then progressed in March 2017 with liver metastasis. The first ctDNA detection (T1) was performed in April 2018, and postoperative lumbar spine metastasis occurred during treatment. The second detection (T2) was performed four months later when the disease had continuously progressed. Based on the patient’s genetic detection results, we recommended alpelisib plus fulvestrant treatment. We tracked the *PIK3CA* p.E545K and *TP53* p.E286K mutations and found that their frequency increased from 0.64% and 0.18% to 2.65% and 1.54%, respectively (Figure 7). The change in ctDNA in the patient’s plasma was consistent with the clinical disease progression, resulting in a poor prognosis. A similar situation occurred in patient P219 with late-stage breast cancer, and the *TP53* p.Y220C mutation increased from 0.16% to 0.97%, with a ctF ratio of 2.310. Additionally, patient p311 was diagnosed with HER2+ breast cancer and received neoadjuvant chemotherapy and target therapy. The patient presented with decreased ctF ratio and tumor size during mutation tracking and reached complete clinical response during surgery.

**Figure 8:**
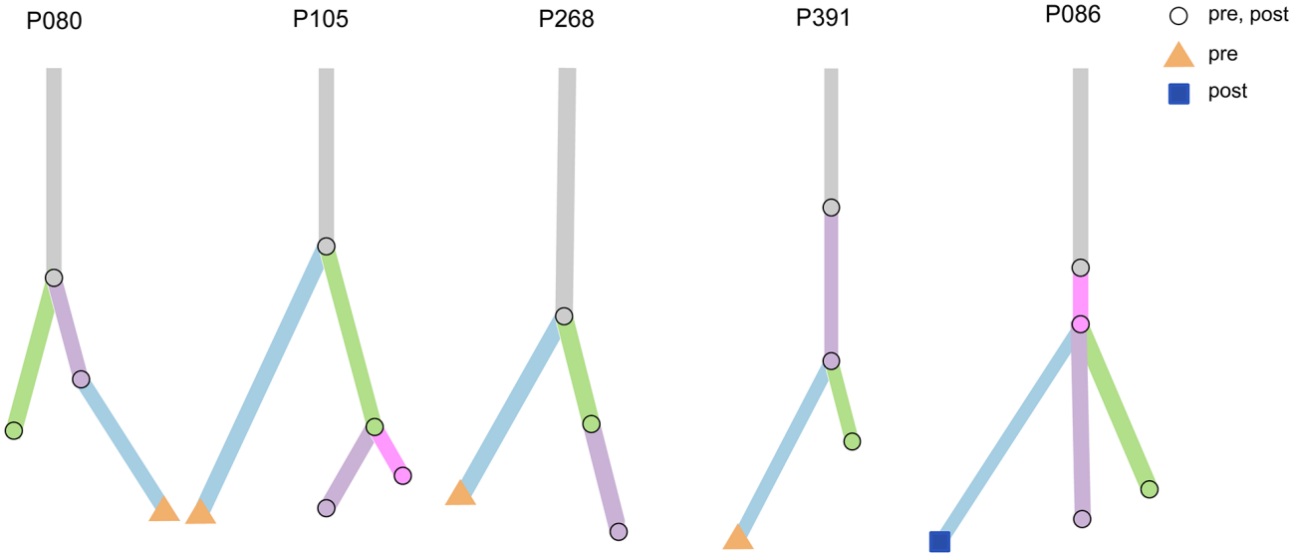
**Clonal analysis of multiple testing in five patients.** Five examples of evolutionary trees. The circles represent mutations present in both pre-treatment and post-treatment; the triangles represent mutations present only in pre-treatment, and squares represent mutations present only in post-treatment.

To further understand the molecular characteristics and clonal evolution in our study samples, clonal analysis was performed for several patients using mutation tracking data. Certain clones were undetectable following treatment in patients P080, P105, P268, and P391. Moreover, ctF values decreased after receiving treatment in these patients, suggesting a decreased tumor mutational burden or disappearance of specific clonal clusters resulting in a relatively better prognosis (Figure 8). However, new emerging clones were observed following treatment in patient P086.

Patient P086 was diagnosed with triple-negative breast cancer (TNBC) in April 2018 and received the first ctDNA detection (T1) before standard neoadjuvant chemotherapy with no significant change observed in the tumor during this period. The patient then discontinued our treatment schedule for four months and came back with a larger mass. The second ctDNA detection was performed in February 2019 (T2), after which an additional four rounds of chemotherapy were performed. No significant change in the tumor was observed during the treatment period. The third ctDNA detection and surgery were performed in May 2019. The newly emerged mutation at T2 was *ERBB2* p.R678Q, which was also detected at T3 with an increase in mutation frequency from 2.81% to 3.9%. The ctF value then increased, while the clone analysis demonstrated an increase in clones. These analyses indicated that the patient exhibited a tendency for disease progression, consistent with the patient’s clinical manifestations. Studies have shown that liquid biopsy is more informative than traditional methods, such as the CA-125 test and imaging, capable of predicting tumor recurrence 7–10 months earlier [26].

Moreover, previous reports indicate that *ERBB2* p.R678Q likely represents an oncogenic mutation [27]. Herein, the cytological study results showed that anti-HER antibody drugs, trastuzumab and pertuzumab, as well as small molecule kinase inhibitors, lenatinib, and lapatinib, elicited inhibitory effects on tumor cells harboring *ERBB2* p.R678Q mutations [28]. Although patient P086 tested negative for HER2 amplification using IHC and FISH, an oncogenic missense mutation in the HER2 subtype was detected using ctDNA, suggesting that HER2-targeted therapy may be an effective follow-up treatment option.

Considering that patient P086 was relatively insensitive to standard neoadjuvant chemotherapy regimen, we next sought to analyze the HRD (homologous recombination deficiency) status of several TNBC patients, including patient P086, to explore optional individualized treatments for TNBC patients with different HRD statuses. The HRD score predicts TNBC or *BRCA1/2*-mutated patient response to DNA damaging neoadjuvant chemotherapy. Patients with an HRD score ≥ 42 were defined as HR deficient and tended to be more sensitive to platinum-containing regimens [29]. Meanwhile, sequencing data analysis revealed that the HRD-sum of patient P086 was remarkably high (Supplementary Table 5), which might be related to the patient’s relatively poor prognosis. Among the five TNBC patients, two were HR deficient, accounting for 40% of the tested patients; the result was consistent with that reported in previous literature (42%) [30]. HRD-positive patients might not benefit from conventional chemotherapy, neoadjuvant chemotherapy, or targeted therapy; however, PARP inhibitors may represent an effective treatment option for these patients [31].

## DISCUSSION

Several studies have recently explored the breast cancer mutational landscape to better understand its genetic complexity [21, 32, 33]. Nevertheless, to the best of our knowledge, ours is the first study to fully elucidate the comprehensive molecular characterization of breast tumors in Chinese women. Specifically, we identified the ctDNA profiles of different pathological subtypes, evaluated the incidence of clinically operable changes, compared the blood and tissue NGS of paired sequenced patients, and evaluated the similarity of blood and imaging evaluations for disease monitoring.

Hence the key findings of our study are as follows: First, ctDNA evaluation effectively captured the landscape of breast cancer genetic alterations, including SNVs, indels, and CNVs. Second, across the entire cohort, genetic alterations were detectable in the blood of >90% of the evaluated patients using the 101 gene panel. Third, the amount of cfDNA extracted from the blood of breast cancer patients and the detected ctF positively correlated with the disease stage. Fourth, differences in the frequency, number, and distribution of gene mutations in the different molecular subtypes of breast cancer were demonstrated.

In our cohort, we found that the *PIK3CA* gene exhibits a higher mutational frequency in TNBC patients, while the *PTEN* gene exhibits higher mutations in HER2+ patients. We also confirmed that TNBC patients exhibited a higher mutation frequency and higher TMB than luminal breast cancer patients, indicating their possible sensitive response to appropriate targeted therapies. Theoretically, tumors with high mutations would generate more proteins that could be recognized by T cells; thus, immunotherapy drugs, such as immune checkpoint inhibitors, may elicit a strong response against these tumors [34]. Therefore, we speculated that the proportion of TNBC patients who benefited from immunotherapy might be higher for the Chinese breast cancer population.

Comparing the test results of paired blood and tissue samples, we found a relatively high consistency in all stages, particularly within patients with late-stage disease. Compared with tissue sampling, ctDNA detection has greater potential for accurately assessing spatial tumor heterogeneity, reflecting the multiple metastatic sites, while also representing a more convenient method for continuous sampling.

*ERBB2* copy number amplification is an essential mutation in breast cancer. Several *ERBB2* mutation-targeting therapies have been proven to be effective for breast cancer patients [35]. Hence, the Food and Drug Administration has approved lapatinib, neratinib, trastuzumab, as well as other small molecule inhibitors and antibody-drug conjugate drugs for the treatment of HER2+ (overexpression/amplification) breast cancer patients. Therefore, accurate detection of *ERBB2* gene amplification is crucial to ensure the effective treatment of breast cancer patients. In our study, the NGS *ERBB2* copy number amplification results for tissue samples were highly consistent with those of IHC and FISH but with poor consistency for blood samples. The low consistency observed in blood samples may be due to high intratumor heterogeneity, which would cause variable amplification of the *ERBB2* copy number in different tumor tissue sites. Additionally, the proportion of examined tumor cells in the tumor tissue samples was higher than 20%; however, only low amounts of DNA were released by the tumor cells into the blood. Hence, the existing NGS analysis platform has certain limitations associated with the analysis of copy number amplification from tumor blood samples.

The present study has several limitations: First, paired sequencing data were only available for a subset of patients. Second, baseline blood collection of all patients was not performed at the same time. Most of our patients have already received multiple treatments; their different treatment options may affect the patient’s mutation spectrum. Third, the prognostic value of frequently altered genes, such as *TP53*, *PIK3CA*, and *ERBB2,* requires further confirmation as our cohort’s overall survival data was not mature. Finally, our study was conducted in a single center with a limited number of patients. It is necessary to verify our results through a multi-center cooperative study.

In our study, patients with decreased ctF ratios appeared more likely to reach complete clinical response or present with higher Miller Payne grades than those with increased ctF ratios after neoadjuvant chemotherapy. Therefore, we postulate that the decreased ctF ratio could reflect the tumor degradation rate and the effectiveness of minimal residual disease clearance. However, further studies with additional statistical analyses are needed to confirm this hypothesis.

## MATERIALS AND METHODS

### Patients and samples

Thirty healthy and 273 breast cancer patients were enrolled at The First Affiliated Hospital of Wenzhou Medical University. All patient-derived specimens and patients’ information were collected and archived under protocols approved by the institutional review board of the First Affiliated Hospital of Wenzhou Medical University. Informed consent was obtained from each patient for the use of their blood and tissue samples. All patients underwent complete tumor staging according to the seventh edition tumor, node, and metastasis (TNM) criteria of breast cancer [36]. A 10-mL sample of whole peripheral blood was collected at initial diagnosis in cfDNA blood collection tubes (Omigen, Hangzhou, China) and was processed for plasma and white blood cells (WBC) within 72 h, resulting in the collection of approximately 4 mL of plasma. The first blood sample from each patient was collected before tissue biopsy/surgery, and some patients had multiple time points of follow-up sampling after therapy. Tumor tissues were collected and either analyzed fresh or were formalin-fixed and paraffin-embedded (FFPE). *Her2* status was tested using IHC. Samples assigned a value of 2+ were further confirmed using FISH. From April 2018 to September 2019, there were 205 cfDNA and 131 tissue/FFPE samples; 104 samples were paired with tissue/FFPE and cfDNA samples.

### DNA extraction

Genomic DNA was extracted from the tumor tissue, FFPE, or WBC using an Omigen extraction kit (Omigen, Inc., Hangzhou, China), followed by fragmentation using an Omigen fragmentation kit (Omigen, Inc., Hangzhou, China). cfDNA was extracted from plasma using an OMInano isolation kit (Omigen, Inc., Hangzhou, China). DNA yield was quantified with Qubit HS DNA (Life Technologies, Carlsbad, CA, USA). All the procedures were performed according to the manufacturer’s instructions.

### Library construction

A DNA library was prepared using an Omigen lib prep kit (Omigen, Inc., Hangzhou, China). Briefly, DNA was end prepared, then ligated with barcoded molecular adaptors, followed by polymerase chain reaction (PCR) amplification to add indexes. Library yield was quantified with Qubit, and the size was measured using a 2100 bioanalyzer (Agilent, Santa Clara, CA, USA).

### Hybrid capture

To ensure minimal coverage, libraries for each pool were prepared according to the Omigen libraries pooling guideline (China patent: 201910515833.4). Libraries were pooled and target enriched using the Omigen 101 genes panel (Supplementary Table 6) and Omigen hybrid capture kit (Omigen, Inc., Hangzhou, China). The captured pool was then amplified using PCR, and quality control (QC) was performed using a 2100 bioanalyzer. The enriched pool was sequenced using Illumina X10 (Illumina, Inc., USA) with paired-end reads and average sequencing depth of 8,000×–13,000× and 40,000×–50,000× for tissue DNA and DMI-tagged cfDNA samples, respectively.

### Sequence data processing and analysis

Somatic single-nucleotide variant (SNV), fusion, and copy number variation (CNV) were analyzed and annotated using the Omigen 101 genes panel (Supplementary Table 6) and Omigen bioinformatics program pipeline including QC, alignment, and variant annotation, followed by filtering. Thirty cfDNA samples from healthy participants were used as background controls.

Exome FASTQ sequences were mapped to the human assembly NCBI build 37 (hg19) using the Bowtie2 alignment software [37]. SAMtools (v0.1.19) was used to convert SAM files to compressed BAM files and sort the BAM files using chromosomal coordinates [38]. PCR duplicates were marked using Picard (v1.131) and subsequently sorted. Marked BAM files were realigned using a Genome Analysis Toolkit (GATK-3.6) at intervals with indel mismatches. GATK was used for performing local realignment for indel calls, and VarDict was used for detecting SNVs [39, 40]. Germline variants were removed by filtering out variants in normal blood samples. Gene annotations and function prediction scores were performed using Annovar, dbSNP build135, 1000Genomes, Polyphen, Avsift, and COSMIC [41–44]. Non-synonymous somatic mutations were identified in the bulk exome sequencing data of five TNBC patients with two or three longitudinal samples. Bulk tumor genomic copy number profiles were estimated from the pair-end exome sequencing depth using the CNV kit [45]. Mutation clusters with only one mutation were excluded from further analysis. The PyClone cluster frequencies were calculated as the mean variant allele frequencies of mutations within each cluster and were visualized using the R package ‘clonevol’ [46, 47].

### HRD score calculation

A portion of sporadic TNBCs and *BRCA1/2*-mutated tumors have DNA repair defects and are sensitive to DNA damage treatment [31]. Based on the loss of heterozygosity (LOH), telomere allelic imbalance (TAI), and large-scale state transition (LST), three independent DNA-based genomic instability measurements were developed. The HRD score represents the arithmetic sum of LOH (number of LOH regions >15 Mb but less than the length of a whole chromosome) + TAI (regions of allelic imbalance that extend to the subtelomere but do not cross the centromere) + LST (breakpoints between regions of imbalance >10 Mb after filtering out regions <3 Mb). HRD scores range between 0 and 100. Tumors with HRD scores ≥42 or *BRCA1/2* mutations were defined as having an HR deficient status. Tumors with an HRD score of <42 that also lacked the *BRCA1/2* mutation were defined as HR non-deficient. The threshold of 42 was selected based on the fifth percentile of HRD scores in tumors with known *BRCA1/2* mutations or methylation status [48, 49]. This process was achieved using a scarHRD software based on whole-exome sequencing data [50].

### Statistical analyses

A *t*-test was performed to compare ctDNA yield, mutation number, and the highest AF between different disease stages and different molecular subtypes. Moreover, their correlation was tested using Pearson’s correlation coefficient. All P values were two-sided, and values <0.05 were considered statistically significant. All statistical analyses were performed using the R language, version 3.6.0.

## Supplementary Material

Supplementary Tables
