# Supplementary material for: Genetic landscape of breast cancer and mutation tracking with circulating tumor DNA in Chinese women
Source: Aging (Albany NY). 2021 Apr 23;13(8):11860–76. doi: 10.18632/aging.202888 (PMC8109076; doi:10.18632/aging.202888)
Supplement: Supplementary Tables [file aging-13-202888-s001.pdf]

SUPPLEMENTARY TABLES

Supplementary Table 1. Basic clinical, demographic, and pathologic characteristics of the breast cancer patient cohort (Number = 273).

Characteristic	Number	Percent (%)
Age (years)		
≤ 60	202	73.99
> 60	71	26.01
Molecular subtype		
Unknown	10	3.66
Luminal A	77	28.21
Luminal B	74	27.11
HER2-positive	67	24.54
Basal-like	45	16.48
TNM[1] stage		
0	7	2.56
I	94	34.43
II	111	40.66
III	31	11.36
IV	30	10.99

[1]Tumor, node, and metastasis according to the 7th edition.

Supplementary Table 2. Next-generation sequencing detection for the prediction of *ERBB2* copy number amplification in breast cancer patients.

Type	TP[3]	FP[4]	TN[5]	FN[6]	Sensitivity	Specificity	Accuracy
TS1	30	1	87	5	0.86	0.99	0.95
CF2	3	5	141	53	0.054	0.97	0.71

[1]Tissue; [2]cfDNA (circulating tumor-derived DNA); [3]true positive; [4]false positive; [5]true negative; [6]false negative.

Supplementary Table 3. Consistency between FISH1 and NGS3 methods for the detection of ERBB2 CNV4 in IHC2 2+ samples.

Sample ID	IHC	FISH	NGS
WY00105	2+	+	+
WY00287	2+	-	-
WY00289	2+	+	+
WY00313	2+	-	-
WY00314	2+	-	-
WY00325	2+	-	-
WY00371	2+	-	-
WY00378	2+	-	-
WY00381	2+	-	-
WY00417	2+	-	-
WY00427	2+	+	+
WY00448	2+	+	+

Supplementary Table 4. Patient information from multiple ctDNA tests.

ID	Age	Stage	Molecular type	Treatment	During treatment	Tumor size after treatment (cm)	ctF ratio	Note
P268	69	III A	luminal B	Epirubicin,Cyclophosphamide,Doxorubicin,Docetaxel,Trastuzumab	/	3.4	0.615	
P164	51	III C	luminal B	Cyclophosphamide,Epirubicin,Surgery	/	0.4	0.182	
P080	61	IV	Her2+	Docetaxel,Trastuzumab,Surgery	3.1	0.5	0.04	
P105	47	III C	Her2+	Cyclophosphamide,Doxorubicin,Docetaxel,Trastuzumab,Surgery	/	0.5	0.423	
P269	50	I	luminal B	Cyclophosphamide,Epirubicin,Docetaxel,surgery	1.8	1.3	0.106	
P221	63	III C	luminal B	Doxorubicin,Cyclophosphamide,Docetaxel	/	2	0.075	
P107	46	II B	Her2+	Cyclophosphamide,Epirubicin,Docetaxel,Trastuzumab,Surgery	/	2.5	0.444	
P321	35	II B	luminal B	Cyclophosphamide,Epirubicin	2.3	2.1	0.359	
P272	45	III A	luminal B	Epirubicin,Cyclophosphamide	/	0.8	0.588	
P311	45	III C	TNBC	Cyclophosphamide,Doxorubicin,Docetaxel,Carboplatin	/	1.6	0.899	
P391	35	II A	TNBC	Cyclophosphamide,Epirubicin,Docetaxel,Carboplatin	/	2.7	0.101	
P112	69	IV	luminal A	Epirubicin,Cyclophosphamide,Gemcitabine,Cisplatin,Nedaplatin,Docetaxel,Zoledronic acid,Letrozole,Surgery	/	4	4.141	Metastasis
P219	39	IV	luminal B	Cyclophosphamide,Epirubicin,Toremifene,Carboplatin,Docetaxel,Surgery	/	4.2	2.310	relapse
P086	44	II B	TNBC	Carboplatin,Cyclophosphamide, Epirubicin,Docetaxel,Surgery	2.6	2.6	32.500	Resistant
P188	55	II B	luminal B	Doxorubicin,Cyclophosphamide,Docetaxel	/	0.9	9.545	

Supplementary Table 5. HRDa score of five TNBC patients.

	HRD-LOH[b]	HRD-LST[c]	HRD-TAI[d]	HRD-sum
WY00086	16	24	34	74
WY00249	2	4	8	14
WY00254	14	22	13	49
WY00330	15	14	12	41
WY00086	16	24	34	74
WY00385	10	11	5	26

[a]Homologous recombination deficiency; [b]Loss of heterozygosity; [c]Telomere allelic imbalance; [d]Large-scale state transition.

Supplementary Table 6. Omigen 101 genes.

AKT1	CDKN2A	GNAS	NF1	RICTOR
AKT2	CRLF2	HNF1A	NFE2L2	RIT1
ALDOA	CTNNB1	HRAS	NOTCH1	RMRP
ALK	DDR2	IDH1	NOTCH2	RNF43
APC	DNMT3A	IDH2	NRAS	ROS1
AR	EGFR	JAK2	NTRK1	RUNX1
ARAF	ERBB2	KDR	NTRK3	SF3B1
ARID1A	ERCC2	KEAP1	PALB2	SMAD4
ATM	ESR1	KIT	PDGFRA	SMO
BCOR	ETV6	KRAS	PDGFRB	SRSF2
BLM	EZH2	LEPROTL1	PIK3CA	STAG2
BRAF	FBXW7	MAP2K1	PIK3R1	STK11
BRCA1	FGFR1	MAP2K2	PMS2	TBC1D12
BRCA2	FGFR2	MAPK1	PTEN	TERT
BRIP1	FGFR3	MET	PTPN11	TET2
CCND1	FLT3	MSH2	RAF1	TP53
CCND3	FOXA1	MTOR	RB1	TSC1
CDH1	GATA3	MYC	RET	TSC2
CDK4	GNA11	NBN	RHEB	U2AF1
CDK6	GNAQ	NEAT1	RHOA	VHL
				ZNF143
